# Targeting CDK5 in Astrocytes Promotes Calcium Homeostasis Under Excitotoxic Conditions

**DOI:** 10.3389/fncel.2021.643717

**Published:** 2021-11-01

**Authors:** Luisa Fernanda Toro-Fernández, Juan Camilo Zuluaga-Monares, Ana María Saldarriaga-Cartagena, Gloria Patricia Cardona-Gómez, Rafael Posada-Duque

**Affiliations:** ^1^Instituto de Biología, Facultad de Ciencias Exactas y Naturales, Universidad de Antioquia, Medellín, Colombia; ^2^Área de Neurobiología Celular y Molecular, Grupo de Neurociencias de Antioquia, Universidad de Antioquia, Medellín, Colombia

**Keywords:** astrocyte, calcium homeostasis, CDK5, excitotoxicity, synaptic protein, hippocampus

## Abstract

Glutamate excitotoxicity triggers overactivation of CDK5 and increases calcium influx in neural cells, which promotes dendritic retraction, spine loss, increased mitochondrial calcium from the endoplasmic reticulum, and neuronal death. Our previous studies showed that CDK5 knockdown (KD) in astrocytes improves neurovascular integrity and cognitive functions and exerts neuroprotective effects. However, how CDK5-targeted astrocytes affect calcium regulation and whether this phenomenon is associated with changes in neuronal plasticity have not yet been analyzed. In this study, CDK5 KD astrocytes transplanted in CA3 remained at the injection site without proliferation, regulated calcium in the CA1 hippocampal region after excitotoxicity by glutamate in *ex vivo* hippocampal slices, improving synapsin and PSD95 clustering. These CDK5 KD astrocytes induced astrocyte stellation and neuroprotection after excitotoxicity induced by glutamate *in vitro.* Also, these effects were supported by CDK5 inhibition (CDK5i) *in vitro* through intracellular stabilization of calcium levels in astrocytes. Additionally, these cells in cocultures restored calcium homeostasis in neurons, redistributing calcium from somas to dendrites, accompanied by dendrite branching, higher dendritic spines and synapsin-PSD95 clustering. In summary, induction of calcium homeostasis at the CA1 hippocampal area by CDK5 KD astrocytes transplanted in the CA3 area highlights the role of astrocytes as a cell therapy target due to CDK5-KD astrocyte-mediated synaptic clustering, calcium spreading regulation between both areas, and recovery of the intracellular astrocyte-neuron calcium imbalance and plasticity impairment generated by glutamate excitotoxicity.

## Introduction

In the last few decades, the role of astrocytes as a type of support cell has expanded to numerous other functions, including formation and regulation of synapses, participation in ion homeostasis, control of the amount of energy delivered to neurons, balance of neurotransmitter levels, and regulation of blood flow to the central nervous system (CNS) (Pekny et al., 2014; Khakh and Sofroniew, 2015). Among these roles, astrocytes participate in crosstalk with neurons that have been described as the “tripartite synapse,” which is composed of a presynaptic terminal, a postsynaptic spine, and an astrocytic process (Araque et al., 1999). Through this complex, astrocytes are capable of modulating neuronal activity by taking up glutamate from the synaptic cleft and releasing the neuronal precursor of glutamate, glutamine. The release of neuronal glutamate favors the increased production of astrocytic glutamine. Thus, the glutamate-glutamine shuttle sustains glutamatergic transmission (Verkhratsky and Nedergaard, 2014).

The regulation of glutamate metabolism by astrocytes requires calcium signaling. Calcium signaling acts as the messenger in this ‘crosstalk’ between astrocytes and neurons. More specifically, astrocytes can sense extracellular glutamate concentrations through calcium waves, thereby leading to the release of gliotransmitters (e.g., adenosine triphosphate, ATP, D-serine, and glutamate, Glu), which regulate synaptic plasticity (Kang et al., 1998; Mothet et al., 2005). The glutamate overstimulation that occurs during excitotoxicity induces a drastic change of intracellular calcium concentrations from 0.2 to 1 μM through a mechanism of extracellular calcium entry or release from the intracellular stores in neurons (Pekny and Nilsson, 2005). However, this calcium concentration also increases in the cytoplasm of astrocytes (Cornell-Bell et al., 1990; Dani et al., 1992). Calcium spread through astrocytic processes buffers Ca^2+^ waves from extraneuronal signaling, constituting a compensation system in the CNS. Nevertheless, chronic calcium overload causes astrocyte reactivity through kinase and phosphatase imbalance (Porter and McCarthy, 1996). It is known that pyramidal neurons from the CA1 area in the hippocampus are susceptible to glutamate excitotoxicity, mainly by expression of NMDA receptors, which are necessary to establish communication between CA3 and CA1 (Spruston et al., 1995; Calvo et al., 2015; Francavilla et al., 2019). A selective induction of astrocytic gliosis generates deficits in neuronal inhibition, increased calcium spreading, and glutamine depression (Ortinski et al., 2010). However, the physiologic astrocytes from the CA3 area of the hippocampus maintain calcium balance, protecting neurons from glutamate excitotoxicity caused by ischemia (Sun et al., 2018). It is not yet clear whether the intervention of astrocytes of the CA3 area change the calcium distribution in the CA1 hippocampal region. Elucidating this role is crucial to mimic physiological astrocytic phenotypes as a possible approach for brain tissue recovery under glutamate excitotoxicity.

Calcium dysregulation is associated with astrogliosis in aging and neurological disorders (Verkhratsky, 2019). Moreover, cytoskeletal stable polymerization is involved in astrogliosis and inflammation (Nourbakhsh et al., 2021), thereby implying kinases such as CDK5 (He et al., 2007; García-Matas et al., 2008). Previously, we have proposed that CDK5 knock-down (CDK5-KD) or inhibition is a convenient strategy to induce astrocyte stellation through BDNF release and reverse the cell death induced by glutamate gliotoxicity (Posada-Duque et al., 2015a; Becerra-Calixto et al., 2018). The astrocyte engraftment or transplantation approach is a growing strategy for brain disorders (Lepore et al., 2008; Davies et al., 2011; Chu et al., 2014; Song et al., 2018). Transplantation of CDK5-KD astrocytes in ischemic rats induces neuroprotection, astrocyte recovery, and neurovascular integrity (Becerra-Calixto and Cardona-Gómez, 2016; Becerra-Calixto et al., 2018). Although it is known that neuronal CDK5 silencing induces CA3-CA1 synaptic plasticity (Posada-Duque et al., 2017), it remains unclear whether the transplantation of CDK5-KD astrocytes can benefit the CA3-CA1 calcium signaling and synapse markers.

Specifically, investigating the regulation of astrocytic and neuronal calcium levels is relevant because these levels can change under glutamate excitotoxicity. Therefore, in the present study, we analyzed the role of CDK5 KD astrocytes transplanted into the CA3 area to understand how these cells affect the calcium levels in CA1 in *ex vivo* hippocampal slices treated with excitotoxic glutamate. Moreover, we assessed *in vitro* the neuronal-astroglial calcium regulation by CDK5 inhibition in astrocytes and how both *ex vivo* and *in vitro* conditions may influence synaptic plasticity markers.

## Experimental Procedures

### Animal Procedures for Cultures

Animals were housed and raised in an SPF (specific pathogen-free) vivarium at SIU-University of Antioquia (Medellín, Colombia) on a 12 h dark:12 h light cycle; food and water were provided *ad libitum*. Rats were handled according to Law 84 of 1989 and Resolution 8430 of 1993 from the Colombian regulations, as well as Public Law 99–158, November 20, 1985, ‘Animals in Research,’ from the National Institutes of Health. Neonatal Wistar rats were sacrificed on postnatal days 1–2 (P1–2) to generate primary astrocyte cultures. Embryonic Wistar rats were sacrificed on day 18 (E18) to generate primary neuron cultures.

### Primary Astrocyte Cultures

Postnatal days 1–2 neonates were sacrificed, and the cerebral hippocampus was removed. Tissues were incubated at 37°C for 15 min with DNase solution (Roche, Basel, Switzerland) and 0.25% trypsin (Gibco, Massachusetts, United States), dissociated and resuspended in DMEM containing 10% FBS (Gibco). Astrocytes were cultivated in T75 flasks (Falcon, Corning, United States) and purified by shaking for 24–48 h from 10–13 days *in vitro* (DIV 10–13). Astrocytes at DIV 16 were transduced with shRNAmiR AAV for *ex vivo* experiments. For *in vitro* experiments, 200,000 astrocytes were cultivated on top of sterile coverslips in 12-well plates (Thermo Fisher Scientific).

### Astrocyte Transduction With shRNAmiR AAV

AVV2.5 viral vectors expressing Scr or CDK5 shRNAmiR-pAAV2.5.CMV.hrGFP were obtained from Davidson Laboratory (Children’s Hospital of Philadelphia) as described previously (Piedrahita et al., 2010). Astrocytes at DIV 16 were preincubated with 1 μg/mL polybrene (Santa Cruz); frozen aliquots were thawed, dialyzed, and resuspended using ice-cold DMEM. Then, cultures were transduced with 2 μL AAV 2.5 (10 genomes per mL) at a 10^12^ titer for 4 h at 37°C (adsorption phase), and the medium was subsequently replaced with DMEM containing 10% fetal bovine serum. The cell cultures were kept at 37°C in an incubator with the appropriate CO_2_ and O_2_ ratio for 8 days until DIV 24 before transplantation.

### *In vivo* Transplantation of Astrocytes

Transduced astrocytes were transplanted *in vivo* in male Wistar rats (4–5 months old). The astrocytes were maintained at 4°C under sterile conditions in both the cell culture room and surgery room. Rats were anesthetized using ketamine (90 mg/kg) and xylazine (5 mg/kg) and received a 2–4% isoflurane and 96% oxygen mixture via an inhalation anesthesia machine after injection in the left hippocampus (−1.72 antero-posterior, 1 lateral, and 3.8 depth) with control astrocytes and astrocytes carrying Scr or CDK5 shRNA-miR. Astrocytes transduced with CDK5 shRNA-miR (CDK5-KD) or Scr shRNA-miR (Scr) had green fluorescent protein (GFP) as a reporter. A total of 1.5 × 10^5^ astrocytes/μL was resuspended in DMEM and arranged in the syringe for subsequent transplantation. Injections were performed via a delivery pump with a Hamilton syringe 26 s/2″/2 (Hamilton^®^ Reno, NV, United States) to a maximum volume of 10 μL at a rate of 0.2 μL/min; a 5-min wait was implemented before withdrawal of the syringe. Transduction efficiency has already been standardized by previous group studies. Post-transplantation, the animals were kept alive for 21 days before sacrifice to ensure that the effects induced by CDK5-KD astrocytes were maintained (Becerra-Calixto and Cardona-Gómez, 2016; Becerra-Calixto et al., 2018).

### Preparation of Acute Hippocampal Slices

Acute hippocampal slices were prepared 3 weeks after AAV viral vector injection in male Wistar rats. Briefly, each rat received an overdose of isoflurane and was killed by decapitation. The hippocampi were rapidly removed and sectioned into 350 μm slices on a vibratome (VT-1000S; Leica, Bensheim, Germany). Hippocampi were dissected using oxygenated ice-cold dissection buffer [composed of (mM) 212.7 sucrose, 5 KCl, 1.23 NaH2PO4, 26 NaHCO3, 10 dextrose, 10 MgCl2, and 0.5 CaCl2] and recovered at room temperature in artificial CSF [ACSF; composed of (mM) 124 NaCl, 5 KCl, 1.23 NaH2- PO4, 26 NaHCO3, 10 dextrose, 1.5 MgCl2, and 2.5 CaCl2] (Posada-Duque et al., 2017).

### Calcium Imaging of Hippocampal Slices

Cytosolic calcium was identified in acute hippocampal slices using the calcium probe Rhod-3 (Molecular probes). Rhod-3 AM (acetoxymethyl) was prepared in ACSF containing 0.01% Pluronic F-127 (Invitrogen) to obtain a probe solution containing 100× concentrated powerload (Molecular probes), 5 μM Rhod3 probe, and 250 nM probendecid (Molecular probes) in ASCF (Wang et al., 2014). Hippocampal slices from rats were loaded with the dye in solution for 60 min in a tightly sealed box filled with 95% O_2_ and 5% CO_2_ at 33–35°C, followed by incubation in standard ACSF for 30 min. Slices were then transferred to an imaging chamber on the stage of a microscope and continuously perfused with oxygenated ACSF at a flow rate of 2 mL/min and a controlled temperature of 30°C. Temperature variations were detected by a digital thermometer to avoid overheating (Tokai hit, Inc.).

**FIGURE 1:**
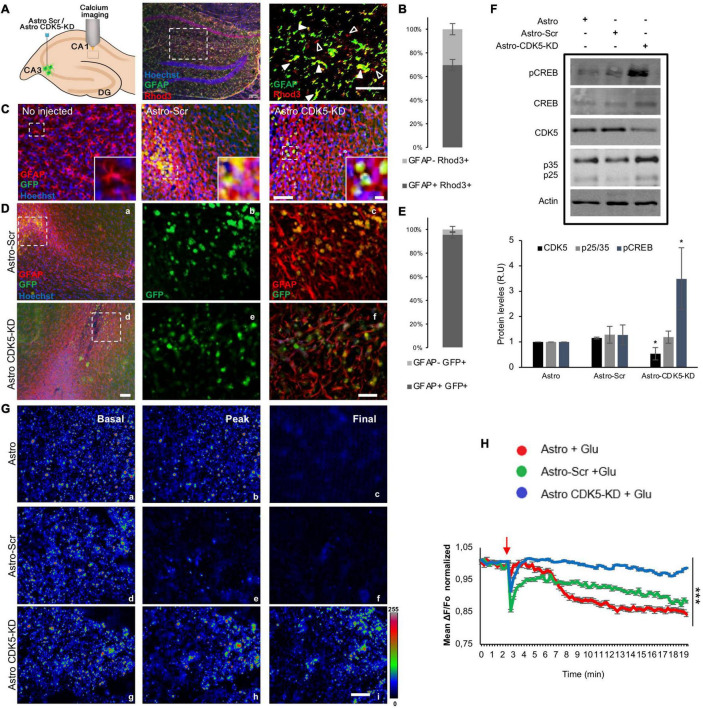
Calcium regulation in the CA1 area by CDK5-KD astrocytes transplanted in CA3. **(A)** A schema illustrating astrocytes injected to the CA3 hippocampus of rats, and calcium signal recorded in CA1. Representative CA1 image of Hoechst (blue), GFAP (pseudocolor-green), Rhod3 (red) staining after intrahippocampal injection of astro, Scr-astro and CDK5-KD astro. Original magnification: ×10 (crop, zoom 1.6), scale bars = 100 μm. White arrowheads indicate double staining of GFAP+ Rhod3+, while empty arrowheads point to GFAP-Rhod3+. **(B)** Percentage of expression of GFAP+ Rhod3+ and GFAP-Rhod3+. **(C)** Representative images of the injection zone in CA3 at 21 days after transplantation or animals no injected. GFAP (pseudocolor in red) and GFP-expressing transplanted cells (green). Magnification: ×10, scale bars = 50 μm; and inset scale bars = 10 μm. **(D)** Immunoreactive GFAP (pseudocolor in red) and GFP-expressing transplanted astrocytes (green). Magnification: ×10, scale bars = 100 μm for a and d. Crop sections were photographed at ×60; scale bars = 20 μm for b,c and e,f. **(E)** Percentage of expression of GFAP+ GFP+ and GFAP-GFP. **(F)** CDK5 silencing and pCREB phosphorylation enhanced by CDK5-KD astrocytes in CA3-CA1. Representative blots and quantification of CDK5, p35/p25 (ratio), and pCREB (ratio to CREB) levels were measured by western blot and were normalized to actin by fluorescence intensity analysis on the bar graph as arbitrary units (RU), *n* = 4; *P < 0.05; Kruskal–Wallis’ test with Dunn’s. **(G)** Rhod3 fluorescence recording in the CA1 area of hippocampal slices at three time points (basal, peak of glutamate addition at minute 3 and final status 20 min later) for Astro (a–c), Astro SCR (d–f) and Astro CDK5 RNAi (h–j) at frames 1 (a,d,g), 12 (b,e,h), and 70 (c,f,i), respectively. **(H)** Kinetics of normalized fluorescent ratio for each treatment (Fo as 1). The fluorescence ratio ΔF/Fo was quantified in CA1 cells treated with glutamate. Each line of the graph shows the average of four independent experiments. Data after Glu stimulus were normalized to basal conditions (Fo). The red arrow indicates the addition of glutamate. Red line indicates Astro, green line indicates Astro-SCR and blue line indicates Astro CDK5-KD. Astro, astrocytes; Astro-SCR, Astrocytes with SCR transduction; Astro-CDK5-KD, Astrocytes with CDK5 RNAi transduction. Magnification ×60; scale bars, 20 μm. The data were relativized to Astro as 100%. ***P < 0.001 ANOVA with Bartlett’s test.

Following incubation with the calcium indicator, the slices were treated with 125 μM glutamate (from 3 to 20 min), and cell imaging was performed using a DSU spinning disk confocal microscope (IX-81 Olympus) coupled to Cell^M system (Olympus) with a xenon light source to avoid phototoxicity, the 60× (NA, 1.42) oil-immersion objective, and a −80°C ORCA camera (Hamamatsu). Live *ex vivo* calcium imaging of CA1 (*sr, striatum radiatum*), preferably from astrocytes, was recorded for 20 min every 60 s until a stable calcium signal was reached (Figures 1A,B). Calcium signals were recorded every 15 s for 3 min as a basal signal and for 17 min after application of the glutamate stimulus. The estimated change in fluorescence intensity over time in CA1 (whole recording field) was determined as a pseudoratio (ΔF/F_0_) using ImageJ software (NIH). This ratio was calculated using the following equation: ΔF/F_0_ = (F – Fbaseline)/(Fbaseline), where F is the indicator fluorescence intensity and F baseline or F0 is the fluorescence intensity before stimulation with glutamate. Images were converted to 8 bits, and the background was subtracted for identification.

### Tissue Immunofluorescence

For this assay, the 350-μm hippocampus slices recorded were immediately fixed using 4% paraformaldehyde (PFA) diluted in cytoskeleton buffer at 37°C for 20 min, and cut to 50 μm in the vibratome (VT-1000S; Leica) from immersion in a 2% agarose gel resin (low melting point) (Sigma). Washes were carried out with 0.1 M PB for 5 min, and then, the tissue was permeabilized using 0.1 M PB and 30% Triton for 30 min. Autofluorescence was removed using 50 mM ammonium chloride (NH_4_Cl) for 10 min. The tissues were subsequently blocked with blocking solution. Later, the cells were incubated for 3 days at 4°C with primary mouse antibodies against GFAP (mouse monoclonal, 1:1000; Invitrogen), PSD95 (mouse monoclonal, 1:500, Calbiochem, CP35) and synapsin (rabbit polyclonal 1:250, Sigma-Aldrich, 574778). Subsequently, they were incubated with Alexa Fluor 647 goat anti-mouse IgG2b (1:750, Invitrogen), Alexa Fluor 488-labeled secondary antibodies (1: 750, Molecular Probes) and Hoechst 33258 (1:5000, Invitrogen). The tissues were fixed to slide plates with FluorSave (Calbiochem). Observation and tissue imaging were performed using a laser scanning confocal microscope (Olympus FV1000) with a 60× oil immersion objective (NA 1.42). XY images consisting of pixel size x/y = 120/125 nm were taken. Images were deconvolved with Huygens Professional 19.10 software probe version (Scientific Volume Imaging).

### Tissue Immunofluorescence Analysis

For each experiment (*n* = 4), 5 slices and 4 fields per slice in the CA1 area of the hippocampus were analyzed to assess morphometric parameters using Image-Pro Plus software (Media Cybernetics, Inc.). The area, perimeter, diameter, width and length of the spots of the markers were determined after filtering by a length not longer than 1 μm and a diameter not longer than 3 μm. The data were analyzed according to the average of each experimental group using multivariate analysis in Python (Python Software Foundation, 1995) with a numpy library (Oliphant, 2006).

Immunoreactivity levels and colocalization of presynaptic (synapsin) and postsynaptic (PSD95) markers and PSD95 with the Rhod3 probe were evaluated with Pearson’s correlation coefficients. Images were deconvolved and converted to 8 bits, and the background was subtracted using Huygens software (scientific volume imaging, BV). Then, the images were calibrated using Image-Pro software, and the “colocalization” tool was used to measure the Pearson correlation coefficient.

The clusters quantification was performed according to Posada-Duque et al. (2017) that relies on determining the synapsin and PSD95 thresholds. A cluster was defined as a particle characterized by diameter (0,2–1 μm) and area (0,02–0,1 μm^2^). The average cluster size was obtained by summing the total area (or perimeter) of each cluster and dividing by the number of clusters.

### Western Blot From Slices and Primary Cultures

The CA1-CA3 region was dissected from frozen hippocampi and homogenized in lysis buffer containing 150 mmol/L NaCl, 20 mmol/L Tris, pH 7.4, 10% glycerol, 1 mmol/L EDTA, 1% NP-40, 100 μmol/L phenylmethylsulfonyl fluoride, 1 μg/mL aprotinin and leupeptin (Sigma Aldrich), and 100 μmol/L orthovanadate. The astrocytes or neuronal cultures were homogenized in lysis buffer and protease inhibitor cocktail mixture (Cytoskeleton Inc.). The lysates were centrifugated at 15,366 × *g* for 5 min. Protein (30 μg) with loading buffer (containing 0.375 mol/L Tris, pH 6.8, 50% glycerol, 10% SDS, 0.5 mol/L DTT, and 0.002% bromophenol blue and heated at 95°C for 5 min) was loaded into each lane in the sodium dodecyl sulfate-polyacrylamide gel electrophoresis (SDS-PAGE; 10%) prepared using a Mini-Protean system (Bio-Rad Laboratories). Next, the proteins were transferred onto PDVF membranes (Amersham) using Mini Trans-Blot Electrophoretic Transfer Cell, at 250 mA for 2 h. The membranes were incubated overnight at 4°C with the following primary antibodies: rabbit anti-CDK5 (1:500; Santa Cruz Biotechnology), rabbit anti-p35 (1:500; Santa Cruz Biotechnology), and mouse anti-phospho-CREB (ser133) (1:500; Cell Signaling Technology), and rabbit CREB 1:500 (Cell Signaling Technology). After several washes, IRDye 800CW goat anti-mouse or IRDye 680 goat anti-rabbit 1:15,000 (LI-COR) were used as secondary probes, and staining was detected using the ODYSSEY Infrared Imaging System (Li-COR, Miami, FL, United States). The band intensities were measured using ImageJ Software (NIH) and normalized to the intensities of control bands (actin, 1:2000; Sigma). The samples from all groups were processed in parallel to minimize inter-assay variation.

### Primary Neuron Cultures

Pregnant Wistar rats were sacrificed in a CO_2_ chamber. Embryos at days 18–19 (E18–19) were extracted and placed in Hank’s Balanced Salt Solution (HBSS, Sigma-Aldrich, Missouri, United States), and then, the neurons were isolated from the cerebral cortex. 70,000 neurons were cultivated on coverslips that had paraffin dots of 0.5⋅2 mm adhered to coverslips that were used to physically separate each cell type. The coverslips were precoated with poly-L-lysine (Sigma-Aldrich). The neurons were maintained in DMEM supplemented with 10% HS in 12-well plates. After 3 h, 10% HS in DMEM was replaced with neurobasal medium (Gibco) supplemented with B-27 and L-glutamine (Sigma-Aldrich). All media used contained penicillin-streptomycin (Gibco). At DIV 3, neuron cultures were treated with AraC to prevent non-neuronal cell proliferation.

### Cyclin-Dependent Kinase 5 Inhibition, Cocultures and Glutamate Treatments

The protocol described by Posada-Duque and collaborators (Posada-Duque et al., 2015a) was modified for use in this study. Astrocytes were subcultivated in 6-well dishes (Falcon) with coverslips for immunofluorescence analysis or in 35-mm glass bottom petri dishes (MatTel Corporation) for calcium recording. Next, 200,000 astrocytes were seeded on coverslips in 12-well dishes containing DMEM and 10% FBS. Coverslips containing neurons were transferred to wells containing astrocytes on DIV 7–8 and maintained in neurobasal medium. When astrocytes reached DIV 22, they were removed from the cocultures, and astrocytic CDK5 was inhibited with 10 μM roscovitine (Rosc) (Calbiochem) for 24 h; then, astrocytes were newly cocultured with neurons. Neurons were never cultured with Rosc. On DIV 17, neurons were removed from the coculture to induce glutamate excitotoxicity with 125 μM glutamate prepared in glutamate buffer for 20 min; neurons were subsequently returned to cocultures. Cells were fixed 1 day after glutamate incubation. For calcium recording, on DIV 24, astrocytes were incubated with glutamate buffer containing the calcium indicator Rhod-2 for 30 min (Thermo Scientific). Next, astrocyte calcium was recorded with 125 μM glutamate to induce excitotoxicity in the coculture for 20 min (for astrocyte calcium records, neurons were not removed from the cocultures). For recording of neurons, the same procedure was performed by seeding 300,00 neurons in recording dishes, and astrocytes were removed for glutamate treatment and calcium recording. Then, astrocytes or neurons were fixed for immunofluorescence.

### Cyclin-Dependent Kinase 5-Knockdown Astrocyte Cocultures and Glutamate Treatment

Scr or CDK5-KD astrocytes were cocultured with neurons (DIV 7) for 10 days before glutamate treatment. At DIV 23 of the astrocyte culture (DIV 17 of the neuron culture), the neuron coverslips were removed for treatment with 125 μM glutamate for 20 min; then, the coverslips were returned to the astrocyte culture for 24 h. Finally, the coculture medium was collected for lactate dehydrogenase (LDH) assays, and astrocytes were fixed for immunofluorescence.

### *In vitro* Cyclin-Dependent Kinase 5 Kinase Assay

At DIV 23, astrocyte cultures were homogenized to determine CDK5 activity. Using 400 μg of total protein, CDK5 was immunoprecipitated with 2 μg IgG antirabbit-CDK5 antibody (Santa Cruz). The immunocomplexes were rotated overnight at 4°C, and protein G Sepharose beads (Sigma) were added and incubated with samples for an additional 4 h at 4°C. The immunoprecipitate (IP) formed by the protein G Sepharose immunocomplexes was resuspended in 25 μL kinase assay buffer (20 mM Tris/HCl pH 7.5, 100 μM sodium orthovanadate, 10 mM MgCl2, 50 mM NaCl, 1 mM DTT, and 1 mM NaF). ATP (0.5 mM) and 6 μM histone-1 (H1) from Calf Thymus Type III-S (Sigma) were added as substrates for CDK5, and the reaction was incubated at 37°C for 30 min. To stop the reaction, 12.5 μL SDS-PAGE loading buffer was added and incubated with samples for 5 min at 95°C. Western blotting assays for phosphorylated H1 (rabbit polyclonal anti-p-H1, 1:500, Millipore), CDK5 (mouse monoclonal anti-CDK5, 1:500, upstate) and p35/p25 (rabbit polyclonal anti-p35 (c-19), 1:250, Santa Cruz) were performed as described previously (Posada-Duque et al., 2015b). IRDye 800CW goat anti-mouse or IRDye 680 goat anti-rabbit 1:15000 (LI-COR) were used as secondary antibodies and detected using an ODYSSEY Infrared Imaging System. The band fluorescence intensities for p-H1, CDK5 and p35/p25 were measured using the Odyssey application software version 3.0 and relativized to CDK5 levels and to the IgG heavy chain intensity.

### Lactate Dehydrogenase Release Assay

Lactate dehydrogenase release from cultures and cocultures was measured using a cytotoxicity detection kit (Roche), and 100 μL of culture medium was collected 1 day after glu treatment (24 DIV). The cytotoxicity was calculated using the following equation: cytotoxicity (%) = [(A-low control)/(high control- low control)]^∗^100, where A was the mean LDH activity measured in medium from two wells that were subjected to test conditions, low control was the LDH released from untreated cultures, and high control was the maximum LDH released from cells treated with 1% Triton X-100 for 24 h.

### Immunofluorescence *in vitro*

Astrocytes at DIV 24 and neurons at DIV 17 were incubated with cytoskeleton buffer for 5 min; then, cultures were incubated with 4% paraformaldehyde (PFA) diluted in cytoskeleton buffer at 37°C for 20 min. After that, the PFA was removed, and cultures were washed with 1× PBS. Autofluorescence was reduced by incubation with 50 mM NH4Cl, and the cells were permeabilized with 0.1% Triton X-100 diluted in 1× PBS for 5 min and blocked with 2.5% FBS (Gibco) for 1 h. Cultures were then incubated with the following primary antibodies overnight at 4°C: anti-CDK5 C8 (rabbit polyclonal, 1:500, Santa Cruz), anti-GFAP (mouse monoclonal, 1:500, Sigma G3893), anti-MAP2 (mouse monoclonal, 1:1000, Sigma M4403), anti-mouse PSD95 (mouse monoclonal, 1:500, Calbiochem, CP35) and anti-synapsin (rabbit polyclonal, 1:500, Calbiochem, 574778). Nuclei were stained with Hoechst 33258 (1:5000, Invitrogen, California, United States), while Alexa Fluor 594- or Alexa Fluor 488-conjugated phalloidin (1:1000, Molecular Probes, Oregon, United States) and secondary antibodies conjugated with Alexa Fluor 594 (1:1000, Molecular Probes) or Alexa Fluor 488 (1:1000, Molecular Probes) were also added. Coverslips containing cells were then mounted on microscope slides using Gel Mount (FluorSave, Calbiochem).

### Image Analysis

Micrographs of neurons were captured with an Olympus IX 81 DSU spinning disk confocal microscope using a 60× objective (NA, 1.42). Astrocyte images were obtained using an Olympus IX 81 epifluorescence microscope with 20× (NA, 0.5) and 60× (NA, 1.42) objectives. For each experiment, 30 astrocytes or neurons (n = 3 duplicate experiments) were counted per treatment group, performed in a blinded mode, for the following morphometric parameters. Fractal analyses (FIJI) were performed on the binary images to calculate the F-actin arborization area in inhibited astrocytes or the GFP area in transduced astrocytes, determined by the convex polygon area obtained connecting the ends of the longest cell extensions. The area and arborization measurements were reported as fold changes and calculated by dividing the values by the value obtained from cells without treatment.

ImageJ software was used to quantify spines and measure dendritic arborization. Spines were counted in F-actin-positive deconvoluted images that had been converted to 8 bits. Images without background were segmented, and quantification was performed after application of the corresponding threshold manual. Spines were counted in the region within 30 microns of the neuronal soma. The Sholl analysis tool was used to measure arborization in MAP2-positive images. The nuclei were quantified in 5 fields (40× magnification), and the mean diameter and blue intensity of each nucleus were quantified using the automatic measurement/spatial trace feature tool. Condensed nuclei were defined within this criterion: a diameter equal to or less than 5 μm and a fluorescence intensity equal to or greater than 120 (AU). The percentage of condensed nuclei was calculated using the following formula:% condensed nucleus = [condensed nucleus/(condensed nucleus + normal nucleus)] ^∗^ 100. The average nuclear areas and diameters were calculated using the tool “spatial trace” (Posada-Duque et al., 2013). The levels and colocalization of presynaptic (synapsin) and postsynaptic (PSD95) markers were evaluated. Pearson’s correlation coefficients for synapsin 1- and PSD95-positive images were obtained using Image-Pro Plus software (Media Cybernetics, Inc.). Images were deconvolved and converted to 8 bits, and the background was subtracted using ImageJ software. Then, the image was calibrated using Image-Pro software, and the “colocalization” tool was used to measure the Pearson correlation coefficient.

### Calcium and Mitochondria Imaging *in vitro*

Intracellular calcium was visualized in primary neuronal and astrocytic cultures using the calcium probe Rhod-2 acetoxymethyl (AM) (Thermo Scientific). Rhod-2 is a cell-permeant AM ester derivative with a positive charge that diffuses into mitochondria through membrane potential-driven uptake (Hoth et al., 1997). Astrocytes were coincubated with Rhod-2 and 2 μM MitoTracker Green (Thermo Scientific) to confirm colocalization between both probes (Supplementary Figure 2C). Neurons or astrocytes were treated with loading buffer in 1× glutamate buffer (vehicle) containing 2.5 mM probenecid acid, 1XPowerLoad^TM^ and 1 μM Rhod-2 AM ester for 15 min at room temperature. Following incubation with the calcium indicator, cultures were treated with 125 μM glutamate, and cell imaging was performed using the 60× (NA, 1.42) oil-immersion objective of the DSU spinning disk confocal microscope (Olympus), coupled to Cell^M system (Olympus) with a xenon light source to avoid phototoxicity, and a −80°C ORCA camera (Hamamatsu). Live cell imaging of astrocytic Ca^2+^ signaling was recorded every 15 s for 10 min after application of the glutamate stimulus. The estimated change in fluorescence intensity over time was determined as described before. For image analysis, the ROIs of somas and processes were determined separately using a manually set ROI in ImageJ. A neuronal or astrocytic process was considered as the protrusion of the cell soma with a length greater than 100 μm, and the ROI was traced manually from the most proximal portion of the soma to the most distal or terminal portion. For this quantification, the branching of the process was not considered (Posada-Duque et al., 2017).

### Statistical Analysis

For *ex vivo* studies, *n* = 4 for each experimental group, and at least four slices per animal were used for calcium, Western blotting and immunofluorescence image analysis. The significance level was ^∗∗∗^*P* < 0.001 based on two-way ANOVA, and the data are expressed as the mean ± SEM. To minimize interassay variation, samples from all experimental groups were processed in parallel. For statistical analysis of the experimental results, the F test was carried out to determine comparisons between group means. Data were compared using one-way ANOVA. GraphPad Prism 7 software (©2017 GraphPad Software, Inc., La Jolla, United States) was used for all statistical analyses.

For *in vitro* assays, data from different treatments (*n* = 3 experiments, 10 cells per experiment) were considered parametric when they met the Shapiro-Wilk normality test. Parametric data were compared using one-way ANOVA followed by Tukey’s test for multiple comparisons. The non-parametric data were analyzed using Kruskal-Wallis and Dunn’s tests for multiple comparisons. Comparisons between two specific groups were performed with Student’s *t* test for parametric data and the Wilcoxon signed-rank test for non-parametric data. The error for multiple testing was corrected using a modified Bonferroni correction. The values are presented as the means ± SEM. The results were considered significant at *P* < 0.05 (95% probability). GraphPad Prism 7 software (©2017 GraphPad Software, Inc., La Jolla, United States) was used for all statistical analyses.

## Results

### Cyclin-Dependent Kinase 5-Knockdown Astrocytes Transplanted on CA3 Cause Calcium Homeostasis in the CA1 Hippocampal Region

Astrocytes allow modulation of neuronal activity, synaptic transmission, and calcium homeostasis (Khakh and Sofroniew, 2015; Nimmerjahn and Bergles, 2015). We previously observed neurological recovery in ischemic rats after CDK5-KD astrocyte transplantation (Becerra-Calixto and Cardona-Gómez, 2016; Becerra-Calixto et al., 2018). In the present study, we determined the cytoplasmic calcium levels at CA1 in *ex vivo* brain slices from 4- to 5-month-old rats transplanted with CDK5-KD astrocytes (Astro-CDK5-KD) in the CA3 area for 3 weeks *in vivo* after glutamate treatment (Figure 1A). We detected the GFP reporter from Scr or CDK5-KD astrocytes in transplanted GFP-treatments groups and observed that the GFP + cells remained in the injection zone, showing double GFAP + GFP + markers (95.5 ± 2.61%) and maintaining a rounded morphology without migration (Figures 1C–E). Endogenous astrocytes from the CA3 area of the transplanted animals showed slight increases in GFAP around the injection zone (Figures 1C,D). We also investigated whether the CDK5-KD astrocytes produce CDK5 silencing in CA3-CA1 after transplantation and whether this event produces CREB phosphorylation, which is involved in a typical synaptic plasticity pathway (Deisseroth et al., 1996; Gutiérrez-Vargas et al., 2016). We found that the transplantation of CDK5-KD astrocytes decreased CDK5 levels and increased the phosphorylation of CREB, compared to untransduced and Scr-transduced astrocytes (Figure 1F). Calcium levels were recorded for 20 min in the CA1 region and, although Rhod3 staining does not have preference for the cell-type, the calcium signal in the recorded zone (*sr, striatum radiatum*) mainly corresponded to GFAP + Rhod3 + cells (69,6 ± 4,7) versus GFAP-Rhod3 + (Figures 1A,B). The calcium signal was described as follows: i. basal, initial conditions (0–2:45 min) (Figures 1Ga,d,g); ii. peak, during glutamate stimulation (3 min) (Figures 1Gb,e,h); and iii. the final interval from 19 until 20 min, corresponding to 16–17 min after glutamate addition (Figures 1Gc,f,i). The estimated change in fluorescence intensity over time was determined as the pseudoratio ΔF/F0 in the whole field. After the excitotoxicity event, calcium signals dropped immediately at minute 3 for all treatments (Figures 1G,H). Although the signal in control astrocytes (Astro) decreased progressively and declined longer and astrocytes transduced with Scr version (Astro-Scr) attempted to recover the signal, astro-CDK5-KD showed calcium signal recovery in CA1 to basal levels (Figures 1G,H). In detail, low calcium signal levels were recovered after astro-CDK5-KD transplantation and remained stable from 4 to 20 min, in contrast with the control cells (Figure 1G). These findings suggest that transplantation of astro-CDK5-KD in CA3 leads to calcium regulation in the CA1 hippocampal region.

### Improvement in Synaptic Markers in CA1 by Astro-Cyclin-Dependent Kinase 5-Knockdown Transplantation in CA3 After Excitotoxicity

**FIGURE 2:**
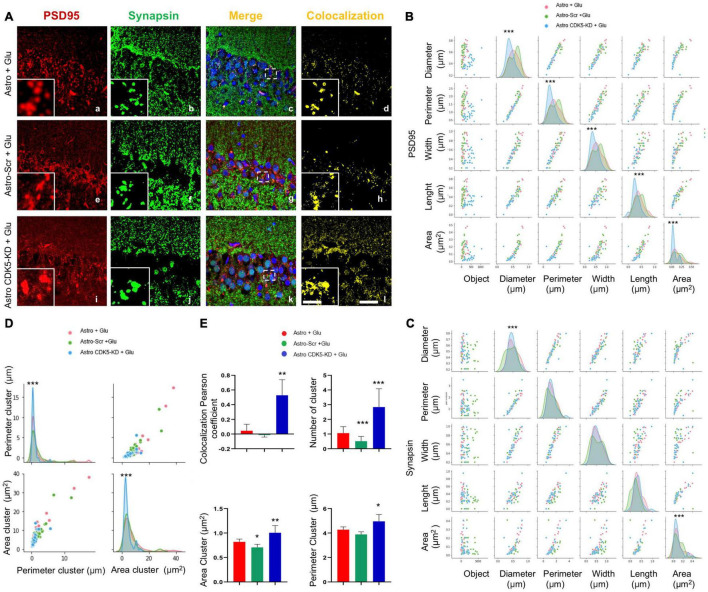
CA3-transplanted CDK5-KD astrocytes improve the formation and characteristics of synaptic clusters in the CA1 area. CA3-transplanted CDK5-KD astrocytes induce synaptic clusters in the CA1 area. **(A)** Representative images of synapsin (green), PSD95 (red), and Hoechst (blue) fluorescence in the CA1 area in hippocampal slices via LCMS and the corresponding image merge and colocalization filter (yellow) for each treatment (Astro, Astro-SCR and Astro-CDK5-KD). Magnification: ×60, scale bars = 10 μm; and inset showing clusters, scale bars = 1 μm. **(B)** Comparison of correlation and distribution of geometric parameters for PSD95 immunofluorescent spots by glutamate (Glu) under three different tissue contexts: Astro (red curve), Astro SCR (green curve) and Astro CDK5-KD (blue curve). **(C)** Comparison of correlation and distribution of geometric parameters for synapsin immunofluorescent spots by glutamate (Glu) under three different tissue contexts: Astro (red curve), Astro SCR (green curve), and Astro CDK5-KD (blue curve). **(D)** Comparison of geometric parameters for synapsin- and PSD95-positive clusters and the correlation and distribution. Astro (red curve), Astro SCR (green curve), and Astro CDK5-KD (blue curve). **(E)** Quantification of synapsin and PSD95 colocalization in clusters by determining the colocalization percentage using Pearson’s coefficient and the number, area and perimeter for each treatment: Astro, Astro SCR and Astro CDK5-KD. Astro, astrocytes; Astro-SCR, Astrocytes with SCR transduction; Astro-CDK5 KD, Astrocytes with CDK5 KD transduction. Representative data are presented as the average ± SEM from *n* = 4 experiments. *Performed in quintuplet. *P < 0.05, **P < 0.01, and ***P < 0.001 using a *t* test.

Astro-CDK5-KD transplantation induced calcium homeostasis under an excitotoxicity event. Therefore, we evaluated whether the stability of calcium signaling corresponded with CA1 (pyramidal layer) synaptic markers (PSD95 and synapsin) after transplantation. PSD95 immunoreactivity did not show changes in the number of clusters (Figures 2A,B); however, all morphometric parameters, such as total area, perimeter, width and length, were increased by astro-CDK5-KD compared with controls (Figures 2A,B). For the synapsin marker, a slight increase in the diameter and area parameters was induced by astro-CDK5 RNAi (Figures 2A,C). Additionally, the intersections at the axis of each parameter showed a linear pattern between samples, supported by scattering, strengthening and homogeneity of data representation (Figures 2B,C). Complementarily, astro-CDK5-KD increased the number of colocalizing synapsin and PSD95 clusters, supported by higher area and perimeter parameters (Figures 2D,E). Additionally, astro-CDK5-KD increased synapsin and PSD95 clustering, as suggested by increased colocalization (Figure 2D). These findings suggest CA1 synapse protection by astro-CDK5-KD transplanted into the CA3 circuit.

**FIGURE 3:**
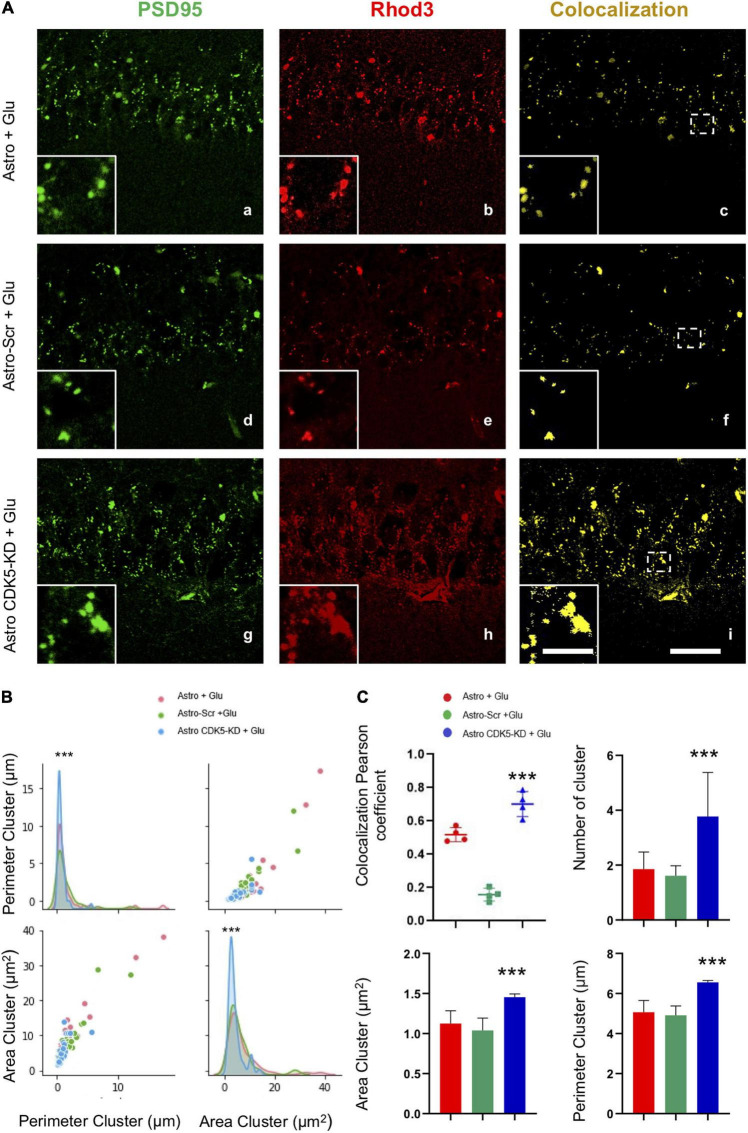
Calcium/PSD-95 clusters recovered in the CA1 area after CDK5 KD astrocyte transplantation in CA3 under excitotoxic conditions. **(A)** Representative images of PSD95 (green) and Rhod 3 (red) fluorescence and binary signal colocalization (yellow) in the pyramidal layer of CA1 area from hippocampal slices imaged via LCMS. Magnification: ×60, scale bars = 10 μm; and inset showing clusters, scale bars = 1 μm. **(B)** Representation of the distribution of the area and the perimeter of the clusters for all conditions Astro (red curve), Astro SCR (green curve) and Astro CDK5-KD (blue curve). **(C)** Quantification of the number of “PSD95 and Rhod3” colocalization clusters using Pearson’s coefficient and the number, area and perimeter for each treatment: Astro, Astro SCR and Astro CDK5-KD. Astro, astrocytes; Astro-SCR, Astrocytes with SCR transduction; Astro-CDK5-KD, Astrocytes with CDK5-KD transduction. The data are presented as the means ± SEMs (*n* = 4), performed in quintuplet. ***P < 0.001 using a *t* test.

The molecular complex at the postsynaptic density is a dynamic structure with direct regulation of calcium signaling, implying synaptic plasticity (Gardoni et al., 2006); therefore, PSD95 and calcium signaling presented an overlapping pattern in the pyramidal layer of CA1 (Figure 3A). Astro-CDK5-KD increased the number of positive colocalizing clusters compared to the control groups (Figure 3C) and led to an increase in the Pearson coefficient (Figure 3C). These clusters showed an increased area and perimeter (Figure 3B), suggesting that astro-CDK5-KD in CA3 recover calcium/PSD-95 in the CA1 area.

### Validation of Cyclin-Dependent Kinase 5 Silencing and Inhibition as an Inducer of Astrocyte Remodeling

**FIGURE 4:**
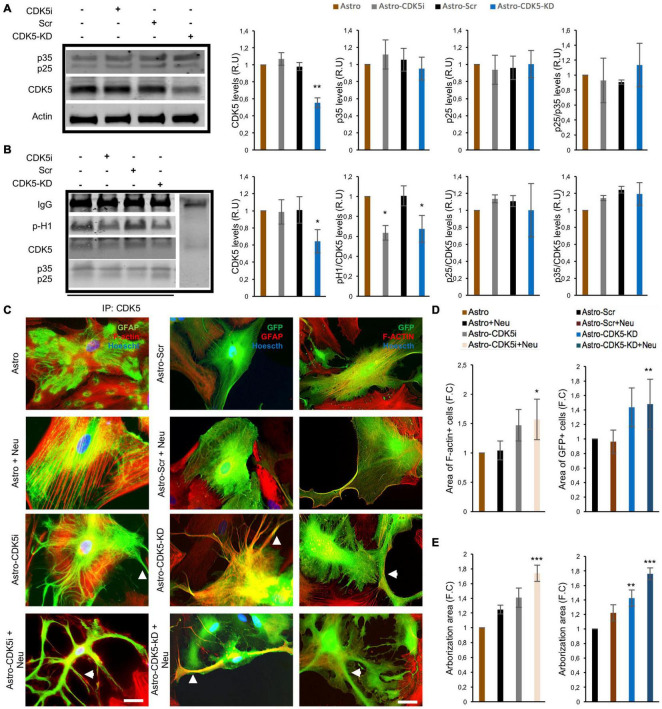
Remodeling of astrocytes induced by CDK5 inhibition and silencing. CDK5 was inhibited for 24 h (astrocytes-CDK5i) or silenced for 11 days (astrocytes-CDK5-KD) in astrocytes. **(A)** Total CDK5 and p35/p25 protein levels relative to actin and analyzed by western blotting. Representative blots are shown as a cropped from different gels for each protein as is indicated in the Supplementary Figure 1A. **(B)** CDK5 kinase activity was assessed for 30 min. Representative CDK5 IP and western blot for p-H1, CDK5, and p35/p25 are shown. Negativity for IgG immunoprecipitation was used as an internal control. Representative blots are shown as a cropped from different gels for each protein as is indicated in the Supplementary Figure 1B. Intensity quantification of p-H1 and p35/p25 is relative to that of CDK5. All values were normalized to control astrocytes and are presented as the means ± SEM. Relative units, RU; *n* = 4. **P < 0.01, *P < 0.05 represent comparisons for Astro and Astro-Scr, respectively. **(C)** Morphological characteristics of astrocytes-CDK5i cocultured with neurons (nuclei, blue; F-actin, red; and GFAP, green) and astrocytes-CDK5-KD cocultured with neurons (nuclei, blue; F-actin, red; and eGFP, green). Micrographs at 60× magnification. Scale bar: 10 μm. *n* = 4. In the images, the arrowhead shows an increased number of processes on the CDK5i and CDK5-KD cells. Fold change data was calculated by dividing the values by the value obtained from cells with astrocytes or Astro-Scr for: **(D)** Area of F-actin+ and GFP+ cells in the CDK5 inhibition and silencing experiment, respectively; and **(E)** the arborization area of the F-actin+ and GFP+ cells was calculated using FracLac (ImageJ). The representative data in **(D,E)** are presented as the means ± the SEMs (*n* = 3), performed in duplicate. *P < 0.05; **P < 0.01; ***P < 0.001.

Neurons control the shape, development and metabolism of astrocytes (Hasel et al., 2017), and CDK5 silencing induces astrocyte remodeling and stellation (Posada-Duque et al., 2015a). Our question was whether pharmacological or genetic silencing of CDK5 could reproduce this effect of protein inhibition and astrocyte remodeling *in vitro*. Therefore, a CDK5 activation assay using an *in vitro* model was performed. CDK5 inhibition with Rosc for 48 h and CDK5 silencing via CDK5-KD for 11 days caused a decrease in histone 1 phosphorylation without any significant change in p35/p25 immunoprecipitation levels, suggesting that CDK5 inhibition or silencing affected CDK5 activity in astrocytes (Figure 4B and Supplementary Figures 1A,B). The specific silencing effect of CDK5 shRNA-miR in astrocytes was confirmed, which showed a decrease of 50% in CDK5 levels (Figure 4A). In contrast, this effect was not detected in the neurons cocultured with astro-CDK5-KD (Supplementary Figure 1C). Then, morphological changes in inhibited or silenced astrocytes were initially determined in coculture with neurons. Astrocytes-CDK5i (Astro-CDK5i) and astro-CDK5-KD or control astrocytes and astro-Scr cocultured with neurons showed higher stellation compared to astrocytes alone (Figures 4C,D). Interestingly, astro-CDK5i and astro-CDK5-KD showed enhanced cellular area and arborizations (stellation) when they were cocultured with neurons (Figures 4C–E). Considering that GFAP underlies astrocyte stellation, GFAP in astrocytes with CDK5 inhibition was detected in cocultures with neurons. Specifically, astro-CDK5i and astro-CDK5-KD distributed GFAP toward the cellular periphery and the end of astrocytic processes, an effect that was enhanced when these cells were cocultured with neurons (Figure 4C). Based on these results, we were able to validate that the astrocytes acquire a stellated morphology following CDK5 targeting.

### Cyclin-Dependent Kinase 5 Inhibition Causes Calcium Redistribution and Homeostasis in Astrocyte Processes

**FIGURE 5:**
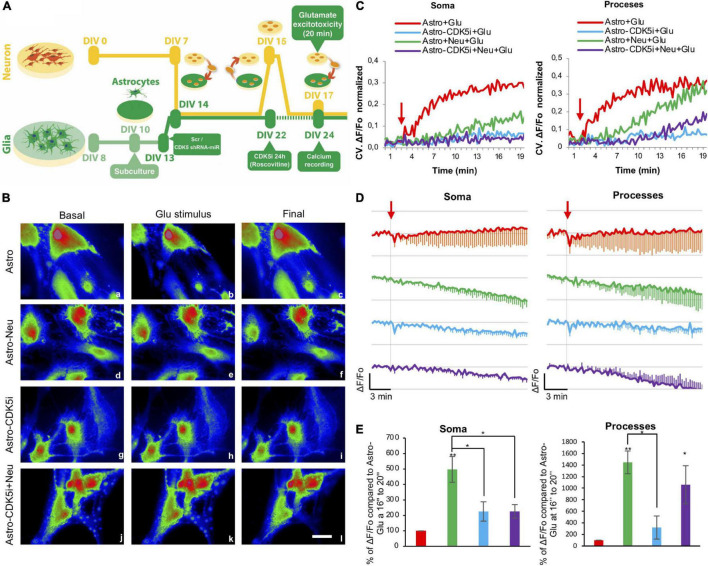
CDK5i causes redistribution and homeostasis of calcium in astrocytic processes. **(A)** Experimental setup of the excitotoxicity model using neuron-astrocyte cocultures. Astrocytes were inhibited with Rosc for 24 h (astrocytes-CDK5i) (DIV 22–23), and neurons (DIV 17) or astrocytes (DIV 24) were treated with 125 μM glutamate. Astrocytes were incubated with Rhod2-AM and treated with glutamate. Astrocytic fluorescence was visualized on DIV 24. **(B)** The images represent the basal conditions (0 min), glutamate stimulus (3 min) and final status (20 min) of the astrocytes loaded with Rhod2 recorded every 15 s for a period of 20 min. The astrocytes were recorded in glutamate (vehicle) buffer for 3 min and treated for 17 min with glutamate. Representative pseudocolored images of neurons loaded with Rho2 (a–l) show the maximum projection 3 min before (a,d,g,j), 15 s after (b,e,h,k) and 17 min after (c,f,i,l) the addition of glutamate. Magnification, 60×; scale bars, 20 μm. **(C)** Quantification of signal fluctuation at each ROI over time determined by calculating the Coefficient of variation (CV) of Rhod-2 signals in soma and processes of astrocytes. The red arrow indicates the addition of glutamate. *n* = 4. **(D)** Kinetic of normalized fluorescence ratio for each treatment (Fo as 1). The fluorescence ratio ΔF/Fo in the soma and processes of astrocytes, astrocytes + Neu, astrocytes-CDK5i and astrocytes-CDk5i + Neu treated with glutamate. Each line of the graph shows the average of four independent experiments. Data after Glu were normalized to basal conditions (Fo). The red arrow indicates the addition of glutamate. **(E)** % of ΔF/Fo compared to Astro-Glu 16″ to 20″, quantified in soma and processes of astrocytes, astrocytes-Neu, astrocytes-CDK5i and astrocytes-CDK5i + Neu treated with glutamate. The data were relativized to astrocytes + Glu as 100%. *P < 0.05; **P < 0.01. ANOVA with Tukey’s test.

Astrocytes act as buffers by recapturing glutamate and calcium during an excitotoxicity process (Wang et al., 2009). Therefore, we determined changes in intracellular calcium levels in the soma and the processes of astrocytes treated with CDK5i using Rhod2-AM on DIV 24 prior to glutamate treatment (Figure 5A). Mitochondrial calcium, sensed by Rhod-2, regulates survival and cell damage pathways (Celsi et al., 2009); specifically, Rhod-2 colocalized with MitoTracker as a specific mitochondrial probe (Supplementary Figure 2C). The calcium in astrocytes in coculture with neurons was registered for 3 min under basal conditions; later, the same field was registered for 20 min with a glutamate stimulus at 180s (Figure 5B). The characteristics of unsynchronized, local and spontaneous calcium transients were investigated by quantifying the magnitude of signal variability in each ROI (soma and processes) over time and calculating the coefficient of variation (CV) of the Rhod-2 signals. Control astrocytes (Astro) with glutamate (Astro + Glu) showed greater fluctuation in the signal over time in soma and processes. This effect was attenuated by coculture with neurons or by CDK5i, which led to a more stable signal (Figure 5C and Supplementary Figures 2A,B). Unexpectedly, in astrocytes-CDK5i cocultured with neurons, there was no attenuated fluctuation of the signal in soma. On the other hand, the estimated change in fluorescence intensity over time was determined as the pseudoratio ΔF/F0. Control astrocytes and astrocytes-CDK5i treated with glutamate showed a small drop in calcium signal and variability in soma and in processes over time (Figures 5D,E and Supplementary Figures 2A,B). Conversely, the astrocytes in coculture under excitotoxicity (astro + Neu + Glu) presented a broad drop and variability of the calcium signal in soma and processes, an effect that was specifically prevented by inhibition of CDK5 in the soma of astrocytes (astro-CDK5i + Neu + Glu) (Figures 5D,E and Supplementary Figures 2A,B). Therefore, astro-CDK5i in coculture prevented the drop in calcium signal levels with respect to control astrocytes (astro + Neu), at least in soma (Figures 5D,E and Supplementary Figures 2A,B), which could be involved in the neuroprotective effect attributed to CDK5 knockdown in astrocytes (Posada-Duque et al., 2015a). These findings suggest that the astro-CDK5i in coculture possibly had the ability to maintain astrocytic calcium homeostasis during excitotoxicity.

### Astrocyte-Cyclin-Dependent Kinase 5 Inhibition Reversed Calcium Loss in the Neuronal Soma and Facilitated Calcium Distribution in Neurites

**FIGURE 6:**
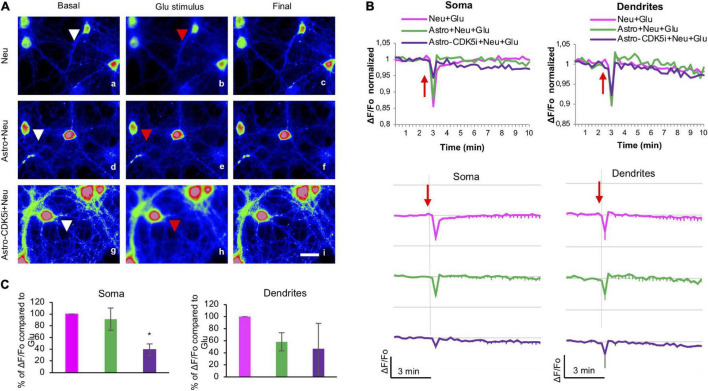
Astrocytes-CDK5i regulate neuronal intracellular calcium levels in the soma and neurites. **(A)** Neurons treated with glutamate were cocultured with astrocytes or astrocytes-CDK5i and incubated with Rhod2-AM, and the fluorescence was visualized at DIV 17. Scale bars, 20 μm. Images depict the initial conditions (0 min), glutamate stimulus (3 min) and final frames (10 min) of Rhod2-loaded neurons (cytoplasmic calcium) registered every 15 s during a 20-min period. Neurons were recorded in glutamate buffer (vehicle) for 3 min and treated with glutamate for 17 min. Representative pseudocolored images of Rho2–loaded neurons (a–i) are shown as maximal projections recorded 3 min before (a,d,g), 15 s after (b,e,h) and 10 min after (c,f,i) the addition of glutamate. Magnification, 60×; scale bar, 20 μm. **(B)** The ΔF/Fo fluorescence ratio was quantified in the soma and neurites of neurons. Each line of the graph shows the mean of four independent experiments. The red arrow indicates the addition of glutamate. **(C)** Percentage of ΔF/Fo fluorescence ratio compared to Neu-Glu at 3″ quantified in soma and dendrites of Astro + Neu and Astro-CDK5i + Neu treated with glutamate. The data were normalized to neurons treated with glutamate, which were set to 100%. *P < 0.05; ANOVA with Tukey’s test.

Astrocytes are related to the calcium response and excitotoxicity in neurons (Matute et al., 2002). Thus, we determined the changes in intracellular calcium levels in the soma and neurites of neurons cocultured with astro-CDK5i using Rhod2-AM at DIV 17 after glutamate treatment. In particular, we examined the average ΔF/F_0_ at 180 s because this time point exhibited peak calcium levels after glutamate treatment (Figures 6A–C). Neurons alone or cocultured with astrocytes showed decreased calcium levels in the soma (Figure 6C and Supplementary Figures 2D,E), whereas neurons cocultured with astrocytes-CDK5i exhibited an approximately 60% restoration of calcium levels (Figure 6C). However, astrocytes-CDK5i maintained decreased calcium concentrations in the neurites, similar to neurons alone or cocultured with astrocytes and glutamate (Figures 6B,C and Supplementary Figure 2B). Based on these findings, astrocytes-CDK5i protect neurons during the acute phase of glutamate excitotoxicity and facilitate calcium dynamics in neurites that are potentially associated with neuroprotection.

### Astrocytes-Cyclin-Dependent Kinase 5 Inhibition Induce Neuroprotective Effects and Enhance Dendritic Arborization

**FIGURE 7:**
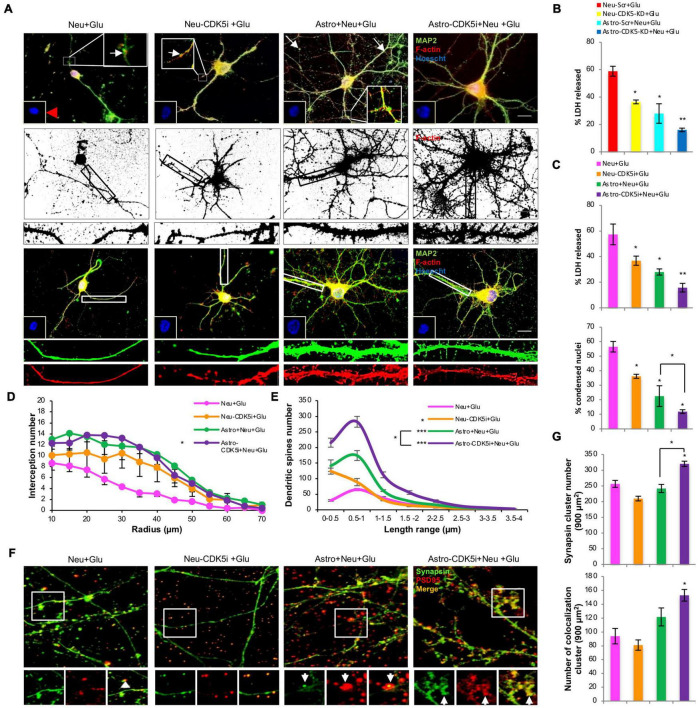
Astrocytes-CDK5i promote neuroprotection, dendritic arborization, dendritic spine formation and synaptic marker recovery in response to glutamate excitotoxicity. **(A)** Morphological characterization showing MAP2 labeled with Alexa Fluor 488 (green), the F-actin cytoskeleton labeled with Alexa Fluor 594-conjugated phalloidin (red, binary segmented images), and nuclei labeled with Hoechst (blue). Magnification, 60×; scale bar, 10 μm; *n* = 4. In the insets, the red arrowhead indicates condensed nuclei, and the white arrowheads mark dendrite degeneration in glutamate-treated neurons. In the cropped image, F-actin (red, deconvoluted and binary segmented images) represents dendritic spines along the MAP2 (green)-positive shafts. **(B)** The percentage of LDH release for the CDK5 silencing experiment. **(C)** The percentage of LDH release and condensed nuclei for the CDK5 inhibition experiment. Condensed nuclei were quantified for each treatment (*n* = 4) compared to neurons treated with glutamate. *P < 0.05 and **P < 0.01; ANOVA with Tukey’s test. LDH release and condensed nuclei were increased in glutamate-treated neurons, and the effect was reversed by silencing **(B)** or inhibition **(C)** of CDK5 in neurons and coculture with control astrocytes (*P < 0.05) and even more powerfully reduced by coculture with astro-CDK5i or astro-CDK5-KD (**P < 0.01); ANOVA with Tukey’s test. **(D)** Sholl analysis of the dendritic arborization in MAP2 images. The number of intersections per ring was significantly increased in neurons with glutamate treatment that were cocultivated with astrocytes-CDK5i compared with neurons treated with glutamate (*P < 0.001). Wilcoxon comparisons with modified Bonferroni correction. **(E)** Spine length distribution for each condition showing the differences in protrusion size. Neurons cocultured with astro-CDK5i showed a greater number of spines with lengths ranging from 0.5–1 μm. Representative data are shown as the averages ± SEM from *n* = 3 experiments. *P < 0.05; ***P < 0.001. **(F)** Synapse characterization showing synapsin labeled with Alexa Fluor 488 (green) and PSD95 labeled with Alexa 594 (red). Magnification, 60×; scale bar, 10 μm; *n* = 4. The arrowheads indicate synapsin and PSD95 puncta and synapsin-PSD95 clustering. **(G)** The number of synapsin and colocalized (synapsin-PSD95) clusters was quantified in 900 μm^2^ (15 cells per experiment in 3 independent experiments per condition were analyzed). Representative data are presented as the average ± SEM from *n* = 3 experiments. *P < 0.05. ANOVA with Tukey’s test.

We determined whether astro-CDK5i and astro-CDK5-KD exerted neuroprotective effects against neuronal excitotoxicity. Neurons that were subjected to glutamate excitotoxicity displayed nuclear condensation, a decrease in nuclear diameter (Figures 7A,C), an increase in LDH release (Figures 7B,C) and loss of dendritic branching, reflected by decreased staining for MAP2 and depolymerization of F-actin compared to the control (Figures 7A,D and Supplementary Figures 3A–D). Despite Rosc-treated neurons (Neu-CDK5i) and control astrocytes prevented these changes, diminishing significantly the LDH release and nuclear condensation neurons showed loss of dendritic structure (Figures 7A–D), it is important to highlight that neurons treated with glutamate and cocultured with control astrocytes showed dendritic toxicity that was consistent with beads in dendrites, an effect that was not detected when neurons were cocultured with astrocytes-CDK5i (Figure 7A). Additionally, astro-CDK5i and astrocytes-CDK5-KD showed reduced LDH release by up to 35% compared with neurons treated with glutamate without astrocytes and did better than control astrocytes (Figure 7C). Additionally, this enhanced neuroprotective effect of astro-CDK5i induced a stronger recovery of dendritic structure (Figure 7D), similar to astro-CDK5-KD in previous reports (Posada-Duque et al., 2015a). These observations indicate that astro-CDK5i exert a greater neuroprotective effect against glutamate-induced excitotoxicity.

### Astrocytes-Cyclin-Dependent Kinase 5 Inhibition Restore Spine and Enhance Synapse Puncta Following Glutamate Excitotoxicity

We investigated whether the neuroprotective effects of astrocytes-CDK5i were associated with the formation of dendritic spines, which are essential for synaptogenesis. We observed that glutamate stimulation in neurons resulted in loss of dendritic spines, evidenced by the decrease in F-actin-rich structures protruding from the dendritic shafts, which were stained for MAP2, compared with the control (Figure 7A and Supplementary Figures 3A,D). These effects were significantly restored by Rosc (Neu-CDK5i + Glu) (Figures 7A,E). Although neurons treated with glutamate and cocultured with control astrocytes showed a significant restoration of the number of dendritic protrusions compared to neurons treated with glutamate, astrocytes-CDK5i displayed a greater number of dendritic protrusions compared with control astrocytes (Astro + Neu-Glu) (Figures 7A,E and Supplementary Figures 3A,E). Furthermore, we analyzed whether the increased number of dendritic protrusions from neurons cocultured with astrocytes-CDK5i generated a shift in the mean protrusion length; the length of functional dendritic spines ranges from 0–2 μm. Neurons treated with glutamate showed a lower number of dendritic protrusions with a decreased length (Figure 7D and Supplementary Figures 3A,E). Moreover, astrocytes *per se* increased the number of functional dendritic spines with lengths ranging from 0–2 μm in neurons, while in glutamate-treated neurons, astrocytes restored spines ranging from 0–0.5 and 0.5–1 μm in length (Figures 7A,E and Supplementary Figures 3A,E). Finally, astrocytes-CDK5i induced a highly significant increase in the number of dendritic protrusions with lengths ranging from 0.5–2 μm (Figures 7A,E and Supplementary Figures 3A,E) in both control and glutamate-treated neurons, indicating that astrocytes-CDK5i enhance the formation of dendritic protrusions with functional sizes that possibly represent mature dendritic spines.

We investigated whether the effects of astrocytes-CDK5i on spines were correlated with changes in synapses; therefore, the number and intensity of colocalized synapsin-PSD95 clusters were analyzed. Control astrocytes and astrocytes-CDK5i increased the number of colocalized clusters in neurons treated with or without glutamate, and this effect was significant in glutamate-treated neurons cocultured with astrocytes-CDK5i (Figures 7F,G and Supplementary Figures 3F,G). Surprisingly, glutamate-treated neurons that were cocultured with astro-CDK5i presented a significant increase in the synapsin cluster number (Figures 7F,G and Supplementary Figures 3F,G). These results show that astrocytes-CDK5i have a positive effect on pre- and postsynaptic clusters and increase the number of synapses.

## Discussion

This study proposes for the first time that CDK5-KD astrocytes transplanted in the CA3 area regulate calcium homeostasis in the CA1 hippocampal area and promote morphological changes in synapses, indicating astrocyte-mediated calcium spreading between both areas. This phenomenon was supported by stellated CDK5-KD astrocytes, which recover the intracellular astrocyte-neuron calcium imbalance and neuronal plasticity detriment generated by glutamate excitotoxicity. This points to CDK5-KD astrocytes as cell therapeutic targets for protecting synapses.

The calcium spreading imbalance at the CA3-CA1 region could be a result of the interaction between endogenous astrocytes and transplanted CDK5-KD astrocytes, which stayed in the injection zone. This result is consistent with previous findings, in which transplanted EGFP-positive astrocytes did not exhibit migration or proliferation (Selkirk et al., 2002; Becerra-Calixto and Cardona-Gómez, 2016). Moreover, the endogenous astrocytes appeared more branched around BDNF positive vessels (Becerra-Calixto and Cardona-Gómez, 2016; Becerra-Calixto et al., 2018), which have been involved in the recovery of LTP synaptic plasticity and CREB phosphorylation in the neuroprotection by CDK5 KD in ischemia (Gutiérrez-Vargas et al., 2016). These findings demonstrate that the viability and permanence of CDK5-KD transplanted astrocytes, specifically in CA3, could promote calcium homeostasis and the phosphorylation of CREB associated with the release of BDNF factor (Posada-Duque et al., 2015a; Becerra-Calixto and Cardona-Gómez, 2016; Gutiérrez-Vargas et al., 2016).

Although the calcium signals detected in the *sr* CA1 are represented mainly by GFAP + Rhod3 + astrocytes, we cannot exclude calcium signal from other types of cells, such as neurons. Glutamate excitotoxicity induces a kinetic pattern of calcium signal characterized by a drop and subsequent recovery by CDK5-KD astrocytes in an *ex vivo* approach. This observation is similar to the pattern observed in neurons and astrocytes that were previously cocultured with astro-CDK5i or CDK5 inhibitor, respectively. Based on our current results, we propose that a prolonged drop in calcium levels triggers cell death. Previously, we have also demonstrated that organoids treated with extracellular vesicles from Alzheimer’s disease samples induce a drop of calcium signal compared to healthy controls, rendering these low levels an indicator of cell damage (Villar-Vesga et al., 2020).

Astrocytes regulate neuronal intracellular Ca^2+^ through glutamate uptake and calcium buffering (Rossi et al., 2007; Sun et al., 2018), protecting the brain parenchyma (Dallérac et al., 2013). Specifically, pyramidal neurons in CA1 and CA3 are particularly susceptible to glutamate excitotoxicity damage. This selective vulnerability is explained by physiological burst firing, which leads to sustained and excessive calcium entry during a brain ischemic attack (Cross et al., 2010). This also suggests that silencing CDK5 avoids abrupt changes in calcium concentrations in astrocytes, possibly by siphoning calcium through the GAP astrocyte network to prevent its overload in pyramidal neurons of the CA1 area (Bellot-Saez et al., 2017), which is also regulated by BDNF from astrocytes (Ohno et al., 2018). Furthermore, the neuroprotection mediated by transplanted astrocytes is supported by the primary astrocyte glutamate transporter, GLT1 (human EAAT2) (Lepore et al., 2008), thereby increasing the uptake of excess glutamate in the parenchyma — which could be enhanced by transplanted CDK5-KD astrocytes.

The ability of CDK5i-astrocytes to prevent changes in Ca^2+^ levels in neurons could possibly be considered a neuroprotective mechanism. More specifically, this phenomenon could be related to Ca^2+^ sequester proteins (e.g., calbindin-D28K), whose overexpression induces neuroprotection against excitotoxic stimuli (Yuan et al., 2013; Xu, 2018). On the other hand, organelles could store calcium to maintain calcium homeostasis. Mitochondria do not serve as large Ca^2+^ “stores” under physiological conditions; however, under conditions of high calcium influx, such as excitotoxicity, mitochondria act as large low-affinity Ca^2+^ reservoirs (Pivovarova et al., 1999). The involvement of mitochondrial Ca^2+^ remains elusive – owing to its complex dynamics and participation in multiple cellular processes. Nevertheless, mitochondria could contribute to this neuroprotective effect by acting as a Ca^2+^ reservoir before triggering mitochondrial pore opening, which would relate to the recovery of the astrocytic and neuronal calcium imbalance induced by CDK5 targeting astrocytes in glutamate excitotoxicity observed in the present study.

Cyclin-Dependent Kinase 5 inhibition in astrocytes induces stellation and reverses calcium loss, supporting the *ex vivo* CA3-CA1 calcium homeostasis effect that has not been sufficiently explored in neuroprotection. While studies have shown that changes in Ca^2+^ levels in astrocytes are involved in the regulation of synaptic plasticity through the release of gliotransmitters such as GABA, ATP, D-serine, and glutamate, whether spatial-time calcium signals generate a specific response in the synapse is still debated (Henneberger and Rusakov, 2010; Henneberger et al., 2010; Di Castro et al., 2011; Panatier et al., 2011; Shigetomi et al., 2012, 2013; Bazargani and Attwell, 2016; Sherwood et al., 2017). The transitory opening of the mitochondrial permeability transition pore is a mechanism for raising Ca^2+^ in microdomains of astrocyte processes (Agarwal et al., 2017). This Ca^2+^ in microdomains exerts an influence on synaptic plasticity because the astrocytic process interacts with the synaptic cleft (Bazargani and Attwell, 2016). In our results, CDK5i-astrocytes prevented the decrease of Ca^2+^ in mitochondria at the time of stimulation with glutamate (peak). This response, to a lesser extent, may be beneficial under certain conditions, since a lower response to glutamate causes less release of Ca^2+^ from mitochondria and therefore less release of gliotransmitters that contribute to neuronal death during excitotoxicity. In addition, CDK5i-astrocytes prevent the loss of the Ca^2+^ levels over time that could affect synaptic plasticity and metabolic activity, which may indicate neuroprotection. The prolonged fall of Ca^2+^ levels in mitochondria and its increase in the soma in a cellular stress event indicates the transition of mitochondrial permeability and the initiation of apoptosis (Alano et al., 2002; MacDonald et al., 2007).

Additionally, we found colocalization of PSD-95 with calcium signal in the pyramidal layer of CA1, which may suggest neuronal calcium to be localized at post-synapses after glutamate excitotoxicity induced by astro-CDK5-KD and a bidirectional astrocyte-neuron balance. Calcium channels are involved in the flux of either presynaptic or postsynaptic terminals (Nett et al., 2002; Sul et al., 2004), and the peri-synapses are covered by astrocytes, implying synaptic neurotransmission and highlighting the mechanism of action of CDK5-KD astrocytes and their specific calcium regulation. CDK5 plays a role in the lethal transfer of calcium from the ER to mitochondria that occurs during neuronal death (Darios et al., 2005). These mechanisms could be part of the explanation of how CDK5 targeting on astrocytes recovered and maintained stable calcium levels after excitotoxic glutamate treatment. However, it will be necessary to go deeper into how the organellar and interorganellar loss of homeostasis in the CA1 cell population is compensated by transplantation of CDK5-RNAi astrocytes in the CA3 region.

Our data support the notion that in the same tissue where CDK5-KD stabilized calcium spreading, the synaptic markers synapsin-1 and PSD-95 were also modified, generating larger synaptic clusters (perimeter and area). PSD-95 diffuses rapidly between synapses, and these PSD-95 levels determine synaptic size and strength during synaptic plasticity (Gray et al., 2006; Glantz et al., 2007). Similarly, synapsin retained the same pattern in synapses involved in the modulation of neurotransmitter release and nerve terminal (Fletcher et al., 1991). Glutamate excitotoxicity generates aberrant accumulation of PSD-95 that does not overlap with synapsin (Posada-Duque et al., 2015b; Uribe-Arias et al., 2016). However, CDK5i-astrocytes exhibit an increase in the number of synapsin-PSD95 clusters, indicating that CDK5i-astrocytes potentially regulate pre- and postsynaptic clustering, most likely through gliotransmitters (Lalo et al., 2016).

Finally, our results also suggest that CDK5i-astrocytes promote spine morphogenesis to functional sizes, which might explain the plasticity mechanism underlying motor and neurological recovery induced by astro-CDK5-KD transplantation in post-ischemic rats (Becerra-Calixto and Cardona-Gómez, 2016). The filopodia-type spines are immature and have lengths greater than 2 μm, with the aid of astrocytes during the maturation process, spines shift through different shapes until they reach a width of 0.6 μm and a length of 1 μm (Risher et al., 2014). These spines represent functional spines that have been associated with synaptic plasticity-dependent processes, such as LTP, learning and memory (Bellot et al., 2014).

In conclusion, targeting CDK5 in astrocytes through silencing and inhibition produces calcium homeostasis in the CA3-CA1 under excitotoxic conditions, likely due to intraneuronal and intraastroglial buffering, which protects synaptic connectivity and promotes neuroglial plasticity, strengthening the idea of CDK5-KD astrocytes as a potential cell therapy for recovering balanced synaptic connectivity and protection.

## Data Availability Statement

The original contributions presented in the study are included in the article/Supplementary Material, further inquiries can be directed to the corresponding author/s.

## Ethics Statement

The animal study was reviewed and approved by Comité de Ética para la Experimentación con Animales de la Universidad de Antioquia (CEEA). Record 119, August 18th 2018.

## Author Contributions

LT-F, JZ-M, AS-C, GC-G, and RP-D designed the experiments, analyzed the data, wrote the manuscript, and reviewed and edited the manuscript. LT-F performed *ex vivo* experiments and analyses of research. JZ-M performed *in vitro* (calcium imaging, knockdown and inhibition validation) experiments. AS-C performed *in vitro* (neuroprotection and structural synaptic plasticity) experiments and analyses of research. All authors contributed to the article and approved the submitted version.

## Conflict of Interest

The authors declare that the research was conducted in the absence of any commercial or financial relationships that could be construed as a potential conflict of interest.

## Publisher’s Note

All claims expressed in this article are solely those of the authors and do not necessarily represent those of their affiliated organizations, or those of the publisher, the editors and the reviewers. Any product that may be evaluated in this article, or claim that may be made by its manufacturer, is not guaranteed or endorsed by the publisher.

## Supplementary Material

Supplementary Figure 1 CDK5 inhibition and silencing in astrocytes. CDK5 was inhibited for 24 h with Roscovitine (vehicle; DMSO) or silenced for 11 days with CDK5 shRNA-miR (control, Scr shRNA-miR) in astrocytes. **(A)** Total p35, p25, CDK5, and actin protein levels assessed by western blotting. Unedited blots are shown for each treatment and blots for each protein were cropped from different gels, as indicated in the green dashed square. **(B)** CDK5 kinase activity was assessed and CDK5 IP and blot for p-H1, CDK5, and p35/p25 are shown. Negativity for IgG immunoprecipitation was used as an internal control. Unedited blots are shown for each treatment and blots for each protein were cropped from different gels, as indicated in the green dashed square. CNT, control cells without any treatment. MW, molecular weight label. **(C)** Neurons (DIV 7) cocultured with scr- or CDK5-KD astrocytes (DIV 14) were treated with 125 μM glutamate. Representative blots and quantification of neuronal CDK5 levels were measured by western blot and were normalized to actin by fluorescence intensity analysis on the bar graph as arbitrary units (RU), *n* = 4; ANOVA with Tukey’s test.

Supplementary Figure 2 CDK5i-astrocytes and CDK5i-neurons calcium recording controls (without glutamate). Astrocytes were incubated with Rhod2-AM. **(A)** Quantification of signal fluctuation in each ROI over time was determined by calculating the coefficient of variation (CV) of Rhod-2 signals in soma and processes of astrocytes *n* = 4. **(B)** Kinetics of normalized fluorescence ratio for each treatment (Fo as 1). The fluorescence ratio ΔF/Fo was quantified in the soma and processes of astrocytes, astrocytes + Neu, astrocytes-CDK5i and astrocytes-CDK5i + Neu. Each line of the graph shows the average of four independent experiments. Data after 3 min were normalized to basal conditions (Fo). **(C)** The images represent the basal and final astrocytes (DIV 24) loaded with Rhod2 (red) and MitoTracker (green); yellow indicates the colocalization overlap area of both signals. Magnification, 60×. Astrocytes were incubated with Rhod2-AM and MitoTracker for 30 min before recording and glutamate treatment. **(D)** Neurons cocultured with astrocytes or CDK5i-astrocytes were incubated with Rhod2-AM. The stimulus ΔF/Fo graph shows the mean fluorescence ratio recorded in the first 3 min after glutamate treatment and was quantified in soma and neurites in neurons cocultured with astrocytes and CDK5i-astrocytes and treated with glutamate. **(E)** Quantification of signal fluctuation in each ROI over time determined by calculating the coefficient of variation (CV) of Rhod-2 signals in soma and dendrites of astrocytes. The red arrow indicates the addition of glutamate; *n* = 4.

Supplementary Figure 3 CDK5i-astrocytes promote dendritic arborization, dendritic spines and synaptic markers. **(A)** Morphological characterization showing MAP2 labeled with Alexa Fluor 488 (green), the F-actin cytoskeleton labeled with Alexa Fluor 594-conjugated phalloidin (red, binary segmented images), and nuclei labeled with Hoechst (blue). Magnification, 60×; scale bar, 10 μm; *n* = 4. The insets indicate normal nuclei. In the cropped image, F-actin (red, deconvoluted and binary segmented images) represents dendritic spines along the MAP2 (green)-positive shafts. **(B)** The percentage of LDH release for the CDK5 silencing experiment. **(C)** The percentage of LDH release and condensed nuclei for the CDK5 inhibition experiment quantified for each treatment (*n* = 4) compared to neurons with glutamate. **(D)** Sholl analysis of the dendritic arborization in MAP2 images. The number of intersections per ring was significantly increased in neurons cocultured with CDK5i-astrocytes, based on Wilcoxon comparisons with modified Bonferroni correction. **(E)** Spine length distribution for each condition showing the differences in protrusion size. Neurons cocultured with CDK5i-astrocytes showed a greater number of spines with lengths ranging from 0.5–1 μm. Representative data are shown as averages ± SEM from *n* = 3 experiments. ^∗∗^*P* < 0.01; ^∗∗∗^*P* < 0.001. **(F)** Synapse characterization showing synapsin labeled with Alexa Fluor 488 (green) and PSD-95 labeled with Alexa 594 (red). Magnification, 60×; scale bar, 10 μm; *n* = 4. The arrowheads indicate synapsin and PSD-95 puncta and synapsin-PSD95 clustering. **(G)** The number of synapsin and colocalized (synapsin-PSD95) clusters was quantified in 900 μm^2^ (15 cells per experiment in 3 independent experiments per condition were analyzed). Representative data are presented as the average ± SEM from *n* = 3 experiments. ^∗^*P* < 0.05. ANOVA with Tukey’s test.

## Abbreviations

ATP: adenosine triphosphate
BDNF: brain-derived neurotrophic factor
CaBP: calbidin protein
CDK5: cyclin-dependent kinase 5
CDK5i: CDK5 inhibition
CNS: central nervous system
DIV: days *in vitro*
ER: endoplasmic reticulum
GFAP: glial fibrillary acidic protein
Glu: glutamate
KD: knockdown
LDH: lactate dehydrogenase
LTP: long-term potentiation
MAP2: microtubule-associated protein-2
NMDAR: NMDA receptor
NVU: neurovascular unit
PFA: paraformaldehyde
PSD95: postsynaptic density-95
ROI: region of interest
Rosc: roscovitine.
