# Supplementary figures and images for: Targeting CDK5 in Astrocytes Promotes Calcium Homeostasis Under Excitotoxic Conditions
Source: Front Cell Neurosci. 2021 Nov 1;15:643717. doi: 10.3389/fncel.2021.643717 (PMC8591049; doi:10.3389/fncel.2021.643717)

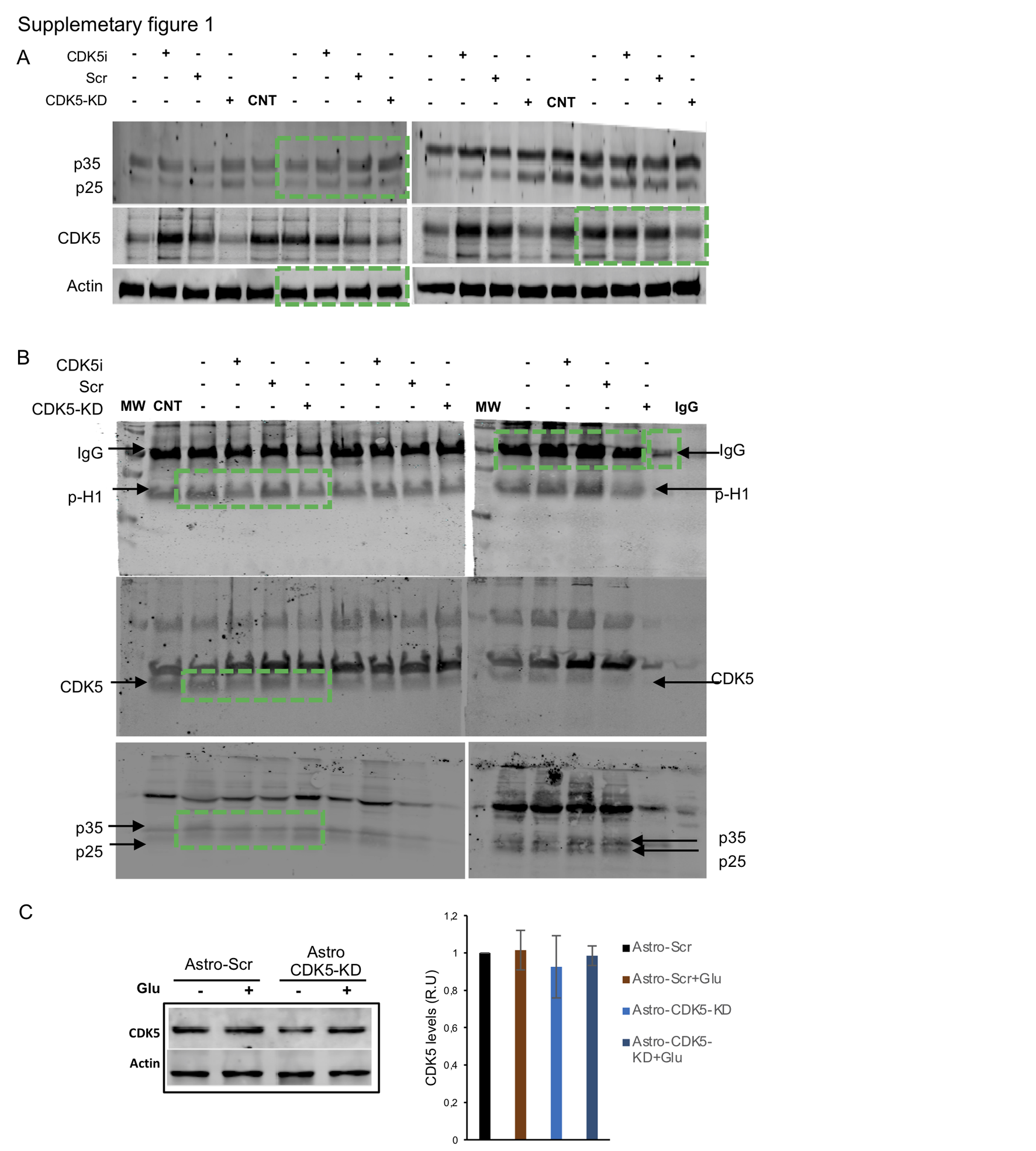

Supplement: Supplementary Figure 1 — CDK5 inhibition and silencing in astrocytes. CDK5 was inhibited for 24 h with Roscovitine (vehicle; DMSO) or silenced for 11 days with CDK5 shRNA-miR (control, Scr shRNA-miR) in astrocytes. (A) Total p35, p25, CDK5, and actin protein levels assessed by western blotting. Unedited blots are shown for each treatment and blots for each protein were cropped from different gels, as indicated in the green dashed square. (B) CDK5 kinase activity was assessed and CDK5 IP and blot for p-H1, CDK5, and p35/p25 are shown. Negativity for IgG immunoprecipitation was used as an internal control. Unedited blots are shown for each treatment and blots for each protein were cropped from different gels, as indicated in the green dashed square. CNT, control cells without any treatment. MW, molecular weight label. (C) Neurons (DIV 7) cocultured with scr- or CDK5-KD astrocytes (DIV 14) were treated with 125 μM glutamate. Representative blots and quantification of neuronal CDK5 levels were measured by western blot and were normalized to actin by fluorescence intensity analysis on the bar graph as arbitrary units (RU), n = 4; ANOVA with Tukey’s test. [file Image_1.tiff]

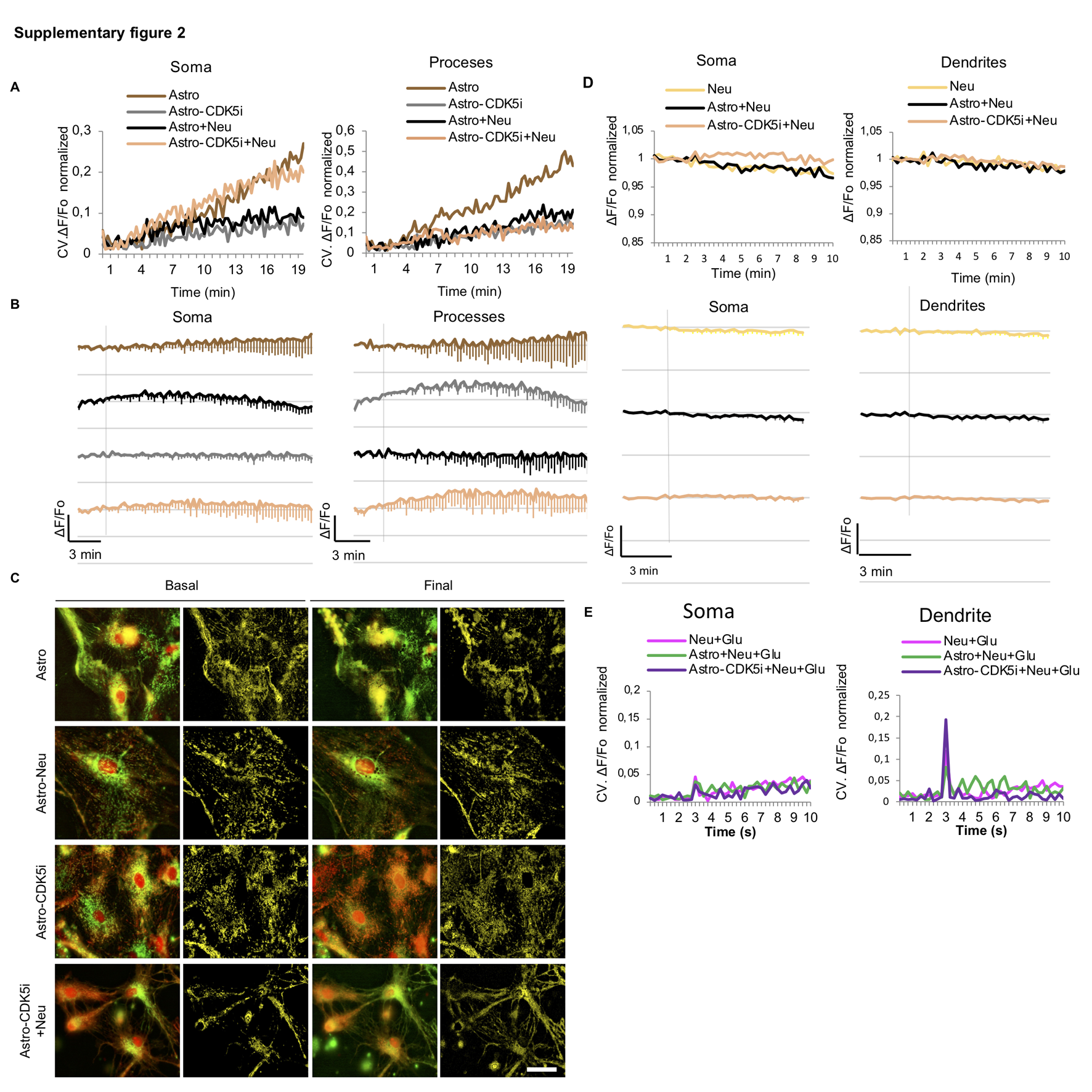

Supplement: Supplementary Figure 2 — CDK5i-astrocytes and CDK5i-neurons calcium recording controls (without glutamate). Astrocytes were incubated with Rhod2-AM. (A) Quantification of signal fluctuation in each ROI over time was determined by calculating the coefficient of variation (CV) of Rhod-2 signals in soma and processes of astrocytes n = 4. (B) Kinetics of normalized fluorescence ratio for each treatment (Fo as 1). The fluorescence ratio ΔF/Fo was quantified in the soma and processes of astrocytes, astrocytes + Neu, astrocytes-CDK5i and astrocytes-CDK5i + Neu. Each line of the graph shows the average of four independent experiments. Data after 3 min were normalized to basal conditions (Fo). (C) The images represent the basal and final astrocytes (DIV 24) loaded with Rhod2 (red) and MitoTracker (green); yellow indicates the colocalization overlap area of both signals. Magnification, 60×. Astrocytes were incubated with Rhod2-AM and MitoTracker for 30 min before recording and glutamate treatment. (D) Neurons cocultured with astrocytes or CDK5i-astrocytes were incubated with Rhod2-AM. The stimulus ΔF/Fo graph shows the mean fluorescence ratio recorded in the first 3 min after glutamate treatment and was quantified in soma and neurites in neurons cocultured with astrocytes and CDK5i-astrocytes and treated with glutamate. (E) Quantification of signal fluctuation in each ROI over time determined by calculating the coefficient of variation (CV) of Rhod-2 signals in soma and dendrites of astrocytes. The red arrow indicates the addition of glutamate; n = 4. [file Image_2.TIFF]

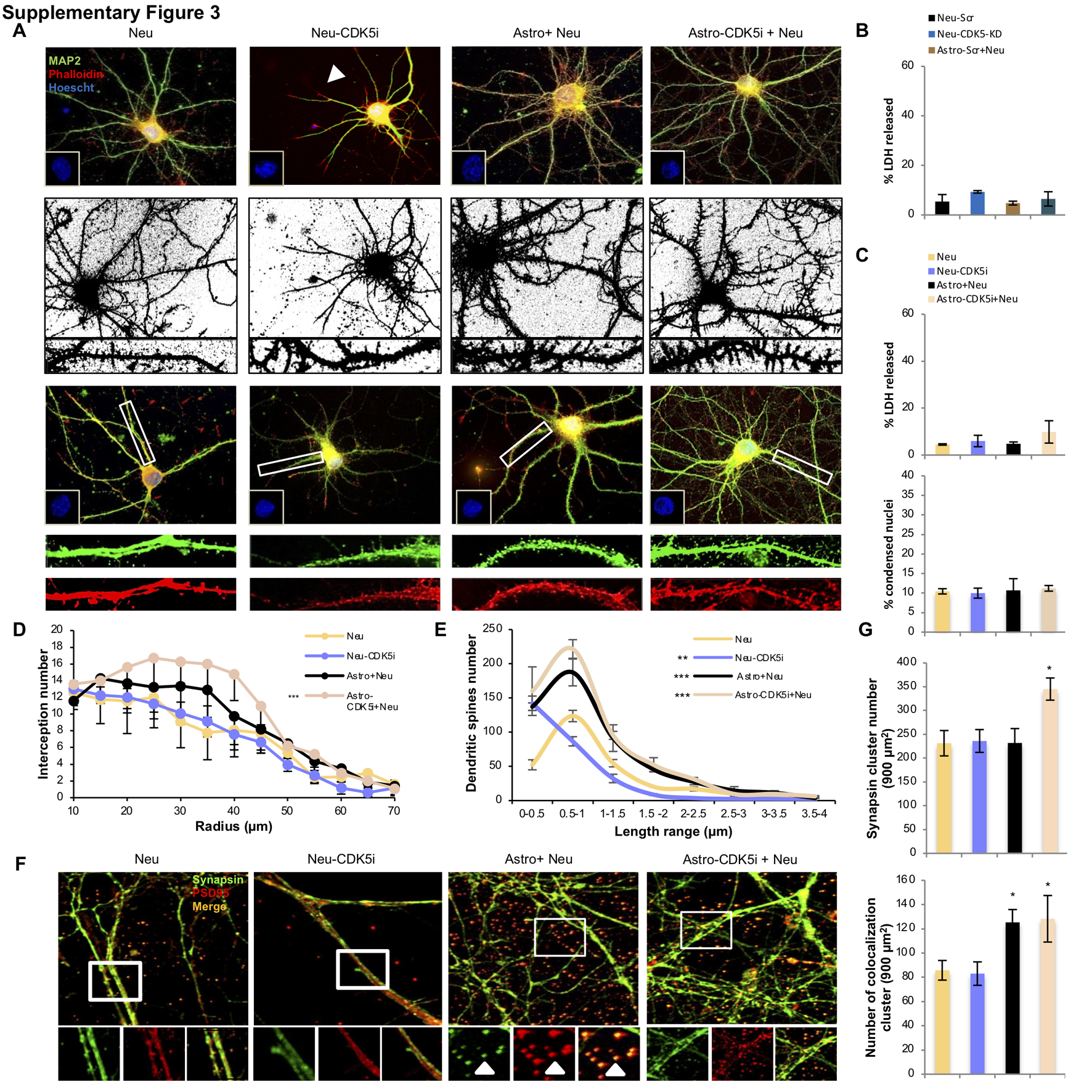

Supplement: Supplementary Figure 3 — CDK5i-astrocytes promote dendritic arborization, dendritic spines and synaptic markers. (A) Morphological characterization showing MAP2 labeled with Alexa Fluor 488 (green), the F-actin cytoskeleton labeled with Alexa Fluor 594-conjugated phalloidin (red, binary segmented images), and nuclei labeled with Hoechst (blue). Magnification, 60×; scale bar, 10 μm; n = 4. The insets indicate normal nuclei. In the cropped image, F-actin (red, deconvoluted and binary segmented images) represents dendritic spines along the MAP2 (green)-positive shafts. (B) The percentage of LDH release for the CDK5 silencing experiment. (C) The percentage of LDH release and condensed nuclei for the CDK5 inhibition experiment quantified for each treatment (n = 4) compared to neurons with glutamate. (D) Sholl analysis of the dendritic arborization in MAP2 images. The number of intersections per ring was significantly increased in neurons cocultured with CDK5i-astrocytes, based on Wilcoxon comparisons with modified Bonferroni correction. (E) Spine length distribution for each condition showing the differences in protrusion size. Neurons cocultured with CDK5i-astrocytes showed a greater number of spines with lengths ranging from 0.5–1 μm. Representative data are shown as averages ± SEM from n = 3 experiments. ∗∗P < 0.01; ∗∗∗P < 0.001. (F) Synapse characterization showing synapsin labeled with Alexa Fluor 488 (green) and PSD-95 labeled with Alexa 594 (red). Magnification, 60×; scale bar, 10 μm; n = 4. The arrowheads indicate synapsin and PSD-95 puncta and synapsin-PSD95 clustering. (G) The number of synapsin and colocalized (synapsin-PSD95) clusters was quantified in 900 μm2 (15 cells per experiment in 3 independent experiments per condition were analyzed). Representative data are presented as the average ± SEM from n = 3 experiments. ∗P < 0.05. ANOVA with Tukey’s test. [file Image_3.TIFF]
